# Silica-calcium phosphate nanoparticles delivering recombinant influenza hemagglutinin DNA can induce long-lasting T cell immune cross-protection in mice

**DOI:** 10.3389/fimmu.2025.1572618

**Published:** 2025-06-03

**Authors:** Xiaoxi Liu, Qingyu Wang, Yuhua Shi, Li Zhan, Lipeng Xu, Jiaru Hui, Kunpeng Xie, Chenxi Li, Chunjiang Li, Weiheng Su, Xianbin Cheng, Yaming Shan

**Affiliations:** ^1^ National Engineering Laboratory for AIDS Vaccine, School of Life Sciences, Jilin University, Jilin, Changchun, China; ^2^ Faculty of Health Sciences, University of Macau, Macao, Macao SAR, China; ^3^ Department of Research and Development, Changchun Gangheng Electronics Company Limited, Jilin, Changchun, China; ^4^ Department of Thyroid Surgery, The Second Hospital of Jilin University, Jilin, Changchun, China; ^5^ Key Laboratory for Molecular Enzymology and Engineering, The Ministry of Education, School of Life Sciences, Jilin University, Jilin, Changchun, China

**Keywords:** influenza virus, hemagglutinin, subunit vaccine, nanoparticles, T-cell immunity

## Abstract

**Introduction:**

Vaccination remains one of the key tools to prevent influenza pandemic. The influenza vaccine induces durable cross-subtype protection through T-cell immunity, demonstrating significant future potential. DNA vaccines are effective in sustaining the expression of antigens, which can trigger T-cell immune responses. Calcium phosphate nanoparticles can also induce T-cell immune responses by assisting in the activation of DC cells by antigens.

**Methods:**

This study developed silica-coated calcium phosphate nanoparticles (226 nm) encapsulating influenza hemagglutinin plasmids (pHAF/pHAG) via polyethyleneimine adsorption. Further analysis of its bioactivity was conducted through experiments.

**Results:**

The nanoparticles demonstrated excellent stability (PDI<0.3 for 7 days), efficient pDNA encapsulation (confirmed by UV), and sustained release (93.14% ± 4.12% at 72 h). DC2.4 cells uptake assays revealed significant antigen-presenting cell internalization (p<0.0001). BALB/c mice were immunized subcutaneously using a prime-boost-boost regimen at two-week intervals. Splenocyte analysis revealed sustained elevation of CD4+ and CD8+ T cell proportions (p<0.05) at 12 weeks post-immunization, suggesting nanoparticle-induced durable T cell immunity. Post-immunization challenge with heterologous H3N2 revealed striking protection: SCPs/pHAF conferred 100% survival, while SCPs/pHAG achieved 66% survival. Notably, SCPs/pDNA immunization significantly reduced lung viral titers versus controls (p<0.05), demonstrating robust cross-subtype protection against lethal infection.

**Discussion:**

This study establishes a significant conceptual framework for advancing the development of DNA-based influenza vaccines with sustained protective efficacy.

## 1 Introduction

Influenza viruses are the causative agent of pandemic influenza. In a typical influenza outbreak year, influenza can cause up to 5 million severe cases and 500,000 deaths (1). To this day, vaccination remains one of the most important ways to prevent influenza pandemics (2).

The development of a universal influenza vaccine, capable of conferring broad-spectrum and durable protection against multiple strains, represents a critical priority in influenza control (3). Notably, T-cell-mediated immunity has emerged as a promising strategy, as it targets conserved viral epitopes and demonstrates the potential for cross-subtype protection, unlike strain-specific humoral responses (4, 5). Crucially, T-cell immunity alone could provide durable protection against lethal viral challenges and inhibit viral transmission (6), highlighting its significant potential for next-generation vaccine development.

The level of T-cell immunity appears to be related to the form of antigen delivery (7–9). Nucleic acid-based vaccines, encompassing both DNA and RNA, demonstrate significant potential in addressing a wide range of indications and diseases (10, 11). Both RNA and DNA vaccines operate by harnessing the host’s endogenous protein synthesis machinery to generate antigens-immunogenic proteins typically expressed on viral or pathogenic cell surfaces (12, 13). It has been shown that delivering antigen into the body in the form of a DNA vaccine can trigger a strong and widespread CD8^+^ T cell response with cytotoxic T cell (CTL) effects (14). What`s more, DNA vaccine production does not depend on live pathogens, thus can be rapidly constructed and manufactured. Besides, DNA vaccine is easy to store and distribute (15), facilitating a rapid response to an influenza pandemic.

Hemagglutinin (HA) is the main glycoprotein of the influenza virus and exists on the surface of the virus as a homotrimer (16). Our former study has prepared a DNA vaccine to immunize mice with H1N1 HA (named HAG and HAF, respectively) demonstrated by multimerization motifs, including the isoleucine zipper of GCN4pII (17) and ferritin (18). The results showed that this vaccine could protect mice against homologous influenza strains and had the potential to serve as an immunogen (19). However, the cross immunity as well as longevity of this vaccine needs to be verified.

A suitable DNA vaccine delivery vehicle can help to trigger a T-cell immune response. Inorganic nanoparticles can be one of the ideal choices for delivering nucleic acids, they are free from microbial erosion and easy to prepare. Besides, it has low toxicity and good storage stability (20). Whereas, calcium phosphate nanoparticles are usually highly biocompatible and well biodegradable compared to other types of nanoparticles (21), and can assist in antigen-induced higher levels of T-cell immunity by coactivating DCs (22, 23). However, DNA-loaded nanoparticles are susceptible to degradation during intracellular translocation into the nucleus (24). So other materials need to be coated on the particle surface to protect the DNA. Silica has a high degree of biocompatibility (20). Previous researches have proved that silica-coated inorganic nanoparticle can protect DNA vaccines in the nucleus (25–27), and in HIV-related studies, nanoparticles of silica-calcium phosphate (SCPs) encapsulated with CpG dinucleotides exhibited significant DNA vaccine delivery efficiency (28), a property that is expected to assist in triggering the T-cell immune response.

In this study, based on the constructed plasmids pHAF or pHAG, the plasmids were adsorbed on the surface of calcium phosphate nanoparticles using polyethylenimine (PEI). Then using silica as the encapsulating shell to form nanoparticles SCPs/pHAF and SCPs/pHAG (collectively referred to as SCPs/pDNA). The particle size was characterized by Dynamic Light Scattering (DLS) for 7 days to monitor the stability of the nanoparticles by Polymer dispersity index (PDI). SCPs/pDNA were transfected into 293T cells to verify the transfection ability of pDNA in SCPs/pDNA. The uptake efficiency of nanoparticles by antigen-presenting cells was evaluated by DC2.4 cellular uptake assay. Immunization of BALB/c mice was performed to evaluate the level of humoral immunity and long-lasting T-cell immunity induced by the nanoparticles *in vivo*. Cross-protection ability of T-cell immunity was evaluated by challenging heterologous virus. SCPs/pDNA nanoparticles are anticipated to elicit durable T-cell-mediated immunity and confer broad-spectrum protection against diverse influenza subtypes.

## 2 Methods

### 2.1 Cells and animals

Human Embryonic Kidney (HEK) 293T cells and Madin-Darby Canine Kidney (MDCK) cells were cultured using Dulbecco’s modified eagle medium (DMEM; Gibco, NE, USA) containing 10% fetal bovine serum (FBS; Gibco, NE, USA). DC2.4 cells and Raji cells were cultured using Roswell Park Memorial Institute (RPMI; Gibco, NE, USA)-1640 medium containing 10% FBS. The above cells were obtained from the American Type Culture Collection, VA, USA, and all cells were cultured at a temperature of 37°C with 5% CO_2_ conditions.

The experimental animals, BALB/c female mice (6–8 weeks old, weight 18–20 g, SPF), were purchased from Liaoning Changsheng Biotechnology Co. Ltd. All procedures were in accordance with the Regulations on the Administration of Laboratory Animals approved by the State Council of the People’s Republic of China, and all animal experimental protocols were approved by the Laboratory Animal Welfare Ethics Committee of the School of Life Sciences, Jilin University (Approval No. 2023-YNPZSY004).

### 2.2 pDNA design synthesis and characterization

The amino acid sequence of HA (1-522) was derived from A/California/17/2009 (H1N1) (GenBank accession number ACR67189.1), and the transmembrane and cytoplasmic structural domains of the sequence were deleted to improve the solubility and stability of the trimeric HA protein. HA (1-522) was fused via a single-copy flexible linker (GSG) to the amino terminus of ferrtin (5-167) (NCBI accession number WP_000949190) and a histidine tag was added at the carboxyl terminus, and the fusion sequence was named pHAF. In the same way, HA (1-522) was fused to the trimeric motif GCN4pII (RMKQIEDKIEEILSKIYHIENEIARIKKLVGER) at the amino-terminus, and the fusion sequence was named pHAG. The nucleic acid sequence was optimized for back-translation using human-preferred codons. The *BamH1* and *Xho1* cleavage sites were respectively added at the 5’ and 3’ ends of the target fragment, and saved in pET20b vector (GenScript, Jiangsu, China), subcloned into pSecTag2A eukaryotic expression vector (lab storage) by *BamH1* and *Xho1* restriction sites, extracted the plasmid by Rapid Plasmid Bulk Kit and sequenced in Changchun comate (Jilin, China). The plasmids were extracted and stored at -20°C.

### 2.3 SCPs/pDNA preparation and characterization

20 mL of 0.02% PEI solution was added to a 50 mL beaker and stirred at 500 rpm using a DF-101S magnetic stirrer (Changchun Jiyu, Jilin, China). 4 mL of 60 mM CaCl_2_ solution and 4 mL of 30 mM Na_2_HPO_4_ solution, respectively, were dropped into the PEI solution simultaneously at a rate of 2~3 drops/second. After stirring at 500 rpm for 30 min, the solution was transferred to an ep tube. SCPs/pDNA was prepared by slowly adding and thoroughly mixing the pDNA solution at a pDNA to SCPs weight ratio of 1:0, 1:1, 1:2, 1:3, 1:4, or 1:5, respectively, to confirm the feeding ratio of pDNA to SCPs. After standing for 30 min, the liquid in the ep tube was dropped at a rate of 2 to 3 drops/sec into a beaker containing 5 mL of ethanol, 15 μL of ethyl tetraethyl orthosilicate (TEOS), and 75 μL of ammonia, and stirred overnight at 100 rpm. The resulting product was centrifuged (6000 × g for 20 min) to collect SCPs/pDNA. Nanoparticles not encapsulated with pDNA, encapsulated with pHAF, or pHAG were named SCPs, SCPs/pHAF, or SCPs/pHAG, respectively. pDNA blocking was electrophoresed in 1% agarose gels at 100 V for 30 min and observed on a UV illuminator (UVP, Jena, Germany) to determine the pDNA to SCPs input ratio.

### 2.4 Identification of SCPs/pDNA particle size and DNA encapsulation

Hydrodynamic dimensions of SCPs/pDNA were measured by DLS on a Zetasizer Nano ZS90 (Malvern Instruments, Malvern, PA, UK). UV spectra of pDNA, SCPs & SCPs/pDNA were recorded on a UV-2550 UV spectrophotometer (Shimadzu, Kyoto, Japan).

### 2.5 SCPs/pDNA *in vitro* release assay

SCPs/pDNA encapsulating 1 mg of pDNA were resuspended using 5 mL of 1× phosphate buffered saline (PBS) (pH 7.4) and sealed in dialysis bags (MWCO, 100 kD), immersed in 20 mL of PBS solution, and incubated at 37°C at 100 rpm in a constant temperature shaker (ZHWY Inc. China) at 37°C and 100 rpm for incubation. At 0 h, 1 h, 2 h, 3 h, 6 h, 12 h, 18 h, 24 h, 48 h and 72 h, 100 μL of PBS outside the dialysis bag was aspirated and an equal volume of PBS was replenished. The pDNA concentration outside the dialysis bag was detected by a Nano-300 microspectrophotometer (Allsheng Instruments, Zhejiang, China) to extrapolate the amount of pDNA released by SCPs/pDNA.

### 2.6 Cell transfection assay

HEK293T cells were inoculated with 1.0×10^5^ cells/well in 24-well plates. After 24 h of incubation, SCPs/pHAG encapsulating 5 μg of pDNA were added to each well. 48 h of incubation was continued, and the cell culture medium was collected for native polyacrylamide gel electrophoresis (Native-PAGE) and analyzed for proteins expressed in HEK293T cells by western blot.

### 2.7 SCPs/pDNA stability studies

SCPs/pDNA was resuspended in PBS and incubated at 4°C or 37°C, respectively. The PDI size of SCPs/pDNA was detected at 0 d, 1 d, 2 d, 3 d, 4 d, 5 d, 6 d and 7 d to determine the stability under storage conditions and *in vitro* physiological conditions.

### 2.8 DC2.4 cellular uptake assays

SCPs/pDNA were dispersed in 0.5 mL of ethanol at a concentration of 100 μg/mL of pDNA and mixed with an equal volume of freshly prepared 0.5 mg/mL Rhodamine B isothiocyanate (RITC; Sangon Biotech, Shanghai, China)/ethanol solution. After mixing and continuous stirring for 3 h away from light, the RITC-labeled SCPs/pDNA (RITC-SCPs/pDNA) was washed and collected. 6-well plates were inoculated with 1.0×10^5^ DC2.4 cells/well, incubated overnight, and the medium was discarded and fresh medium containing RITC-SCPs/pDNA was added. Cells were washed after 4 h of incubation, fixed in 4% paraformaldehyde, and the mean fluorescence intensity (MFI) was measured using a CytoFLEX flow cytometer (Beckman Coulter, CA, USA) to assess cellular uptake. 4’,6- diamidino-2-phenylindole (DAPI; Genview, FL, USA) stained nuclei after fixation. Fluorescence images were recorded using an IX73 inverted fluorescence microscope (Olympus Corporation, Tokyo, Japan).

### 2.9 BALB/c mice immunization

Female BALB/c mice were randomly divided into three groups (n = 4): SCPs/pHAF, SCPs/pHAG, and PBS (control). SCPs/pDNA was resuspended in PBS, and the concentration was adjusted to 0.5 μg/mL as pDNA, and 200 μL was immunized each time by subcutaneous injection. Three immunizations were performed at two-week intervals. Serum samples were collected at 2-week intervals, heated at 56°C for 30 min to inactivate complement, and stored at -80°C until analyzed.

### 2.10 HA-specific binding antibody titer assay

2 μg/well of A/California/07/2009 H1N1 HA (Sino Biological, Beijing, China) was coated on a 96-well plate and incubated at 4°C overnight. The next day, the plates were washed with PBST containing 3% BSA at 25°C and incubated with bovine serum albumin (BSA) (Solarbio, Beijing, China) at a concentration of 3% for 1 h. 50 μL/well of 10-fold gradient diluted serum samples were added and incubated at 37°C for 2 h. Subsequently, 50 μL/well of Horseradish Peroxidase (HRP)-coupled anti-mouse IgG (Dingguo, Beijing, China) was added and incubation continued for 45 min. After incubation. 100 μL/well of 3,3′,5,5′-tetramethylbenzidine solution (TMB) was added, and the color development was avoided for 15 min, and 50 μL/well of 2M H_2_SO_4_ was used to terminate the color development. The absorbance at 450 nm was recorded using an iMarK™ enzyme marker (Bio-Rad, CA, USA).

### 2.11 Antibody-dependent cell-mediated cytotoxicity assay

Splenic lymphocytes were isolated from unimmunized mice using the Mouse Splenic Lymphocyte Isolation Kit (Solarbio, Beijing, China). Raji cells were inoculated and infected with 100×50% tissue culture infective dose (TCID_50_) of A/California/07/2009 H1N1 virus, and cultured in RPMI1640 serum-free medium for 12 h. The targeted cells were further stained with 0.5 μM carboxyfluorescein succinimidyl ester (CFSE; Invitrogen, USA) and then incubated with immunized mouse serum (1:1000 dilution) for 1 h at 37°C.

Next, effector cells were added to the infected Raji cells at a ratio of 10:1 and incubated at 37°C for 4 h. All cells were stained with propidium iodine (propidium iodine, PI; BioLegend, CA, USA) and analyzed using a CytoFLEX cytometer. The percentage of antibody-dependent cell-mediated cytotoxicity (ADCC) activity was calculated as follows: ADCC% = [(percentage of target cell group lysed - percentage of spontaneous lysed group lysed)/(percentage of positive control group lysed - percentage of spontaneous lysed group lysed)] × 100%, where cells in the target cell group were not lysed. where cells in the spontaneous lysis group were not supplemented with immunized mouse serum and cells in the positive control group were completely lysed using 0.5% Triton X-100.

### 2.12 Cytokine secretion level assay

Splenic lymphocytes were isolated in immunized mice using the Mouse Splenic Lymphocyte Isolation Kit. The lymphocytes were divided into experimental and negative control groups, and both 3×10^5^/well were added to pre-coated ImmunoSpot plates (Dakewe, Guangdong, China). 10 μg/mL of A/California/07/2009 H1N1 HA protein was added to the wells of the experimental group as a stimulus, and 10 μL of serum-free medium was added to the wells of the negative control group. The plates were incubated at 37°C for 18 h. Cells were removed and biotin-coupled anti-IFN-γ antibody was added and incubated at 37°C for 1 h. Subsequently, streptavidin-HRP was added to the plates for 1 h at 37°C. Finally, 3-amino-9-ethylcarbazole (AEC) solution was used to develop the spots, and the number of spots was counted using an ImmunoSpot analyzer (Cellular Technology Ltd., OH, USA).

### 2.13 Hemagglutination inhibition assay

Serum samples were serially diluted 2-fold and incubated with 4 HA units of A/17/California/2009/38 (H1N1) virus (Changchun BCHT, Jilin, China) for 1 h at 37°C. The serum samples were then incubated for 1 h at 25°C. An equal volume of 0.5% fresh chicken erythrocytes (Solarbio, Beijing, China) was then added to each well and incubated for 45 min at 25°C. The HI titer was defined as the reciprocal of the highest serum dilution that inhibited hemagglutination.

### 2.14 Micro-neutralization assay

MDCK cells were cultured in 96-well plates with DMEM medium (10% FBS and 1% antibiotics) at 37°C and 5% CO_2_ environment. Serum was serially diluted twofold with viral medium (DMEM containing 0.3% BSA), and the dilution (50 μL) was mixed with 100 TCID_50_ of influenza A/17/California/2009/38 H1N1 virus (50 μL) and placed into 96-well plates and incubated for 1 h at 37°C, followed by addition of MDCK cells (1.5 × 10^4^ cells/well) and incubated at 37°C. After 48 h, 50% inhibitory dose (ID_50_) values were measured using visualization of the cytopathic effect (CPE). ID_50_ was defined as the serum dilution that neutralized 50% of the virus.

### 2.15 Measurement of long-lasting T-cell activation levels

12 weeks after the end of immunization, splenic lymphocytes were isolated from immunized mice by the Mouse Splenic Lymphocyte Isolation Kit. Cells were washed once with ice-cold cell staining buffer and suspended in 50 µL of ice-cold blocking solution (TruStain FcX PLUS antibody (BioLegend, CA, USA) in cell staining buffer), and incubated for 10 min on ice. Afterwards, 1×10^6^ lymphocytes were co-incubated for 20 min on ice with anti-CD3-fluorescein Isothiocyanate (FITC), anti-CD4-phycoerythrin (PE) and anti-CD8-allophycocyanin (APC) (BioLegend, CA, USA) were co-incubated on ice for 20 min. The percentage of CD3^+^ CD4^+^ and CD3^+^CD8^+^ lymphocytes were identified by CytoFLEX cytometry (Beckman Coulter, USA) and analyzed with FlowJo V.10.1 software.

### 2.16 *In vivo* CTL assay

Lymphocytes from naïve mice were isolated using the Mouse Splenic Lymphocyte Isolation Kit. A portion of the cells were stimulated with 5 μg/mL antigen and incubated with a high concentration of CFSE (10 μM), while unstimulated cells were incubated with a low concentration of CFSE (1 μM). The two portions of cells were mixed in a 1:1 ratio and injected intravenously into immunized mice. Lymphocytes were isolated from naive mice 15 h after injection and analyzed using flow cytometric detection.

### 2.17 Virus challenge

Eight weeks after the end of immunization, mice were immunized three times. Two weeks after the third immunization, mice were intranasally infected with 100 μL of 10×median lethal dose (LD_50_) of A/Hong Kong/2671/2019 (H3N2) virus (Changchun BCHT, Jilin, China) (50 μL per nostril). Body weight changes and survival of mice were monitored for 14 consecutive days after the virus attack. Weight monitoring was terminated when the mice lost ≥25% of their body weight. Survival rates in mice were monitored continuously throughout the study period. The mice were euthanized according to the guidelines of the Laboratory Animal Welfare Ethics Committee of the School of Life Sciences, Jilin University.

### 2.18 Lung viral titer test

One mouse in each group was euthanized on day 4 after infection. Lung tissue was removed and homogenized in 5 mL of PBS. The supernatant was collected for a 10-fold serial dilution, followed by the addition of MDCK cells (1.5 × 10^4^ cells/well) and incubated for 72 h. The TCID_50_ was measured using the visualization of the CPE to determine the pulmonary virus titer in cells.

### 2.19 Histological analysis

Lungs of mice were collected on day 4 after infection, fixed in 4% paraformaldehyde and embedded in paraffin blocks. Tissue sections were dehydrocarbonized with xylene, dehydrated with an ethanol gradient, and immersed in distilled water. Thin 3-μm sections were stained with hematoxylin and eosin (H&E), and the histomorphology of the lungs was observed under light microscope.

### 2.20 Data analysis

All data are expressed as mean ± standard deviation (SD). They were analyzed using GraphPad Prism 8.0 software. Ordinary one-way analysis of variance (ANOVA) groups were used for comparison between groups. Tukey ‘s multiple test was used for statistical analysis of comparative tests. (Descriptions are as follows: *, 0.01<*p*<0.05; **, 0.001<*p*<0.01; ***, 0.0001<*p*<0.001; ****, *p*<0.0001; ns, not statistically significant).

## 3 Results

### 3.1 SCPs can encapsulate pHAF with pHAG

**Figure 1:**
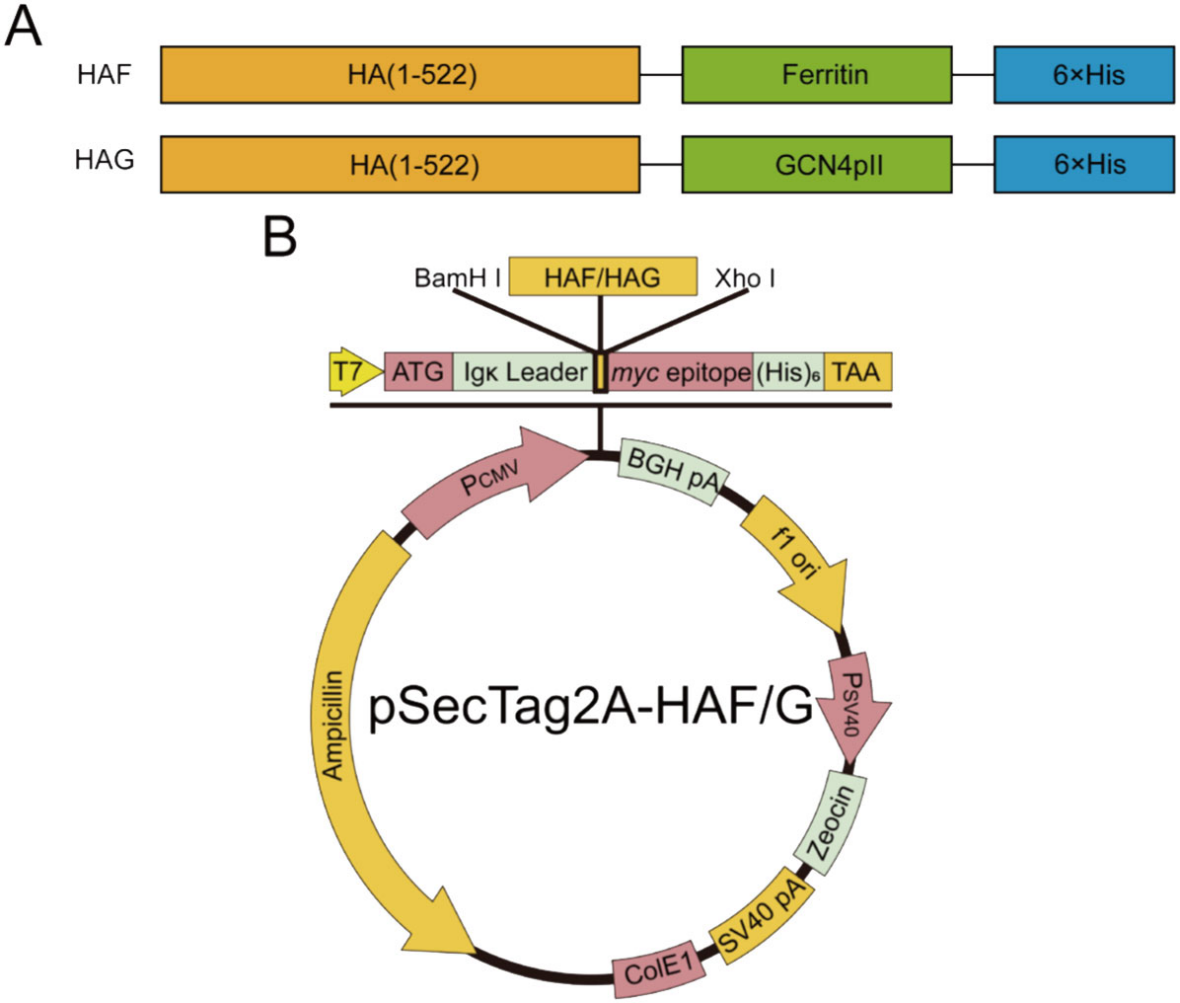
Schematic illustration of nanoparticle construction. **(A, B)** HA was genetically fused to the N-terminus of either ferritin or the trimeric GCN4pII motif, with a C-terminal hexahistidine tag incorporated for purification purposes.

**Figure 2:**
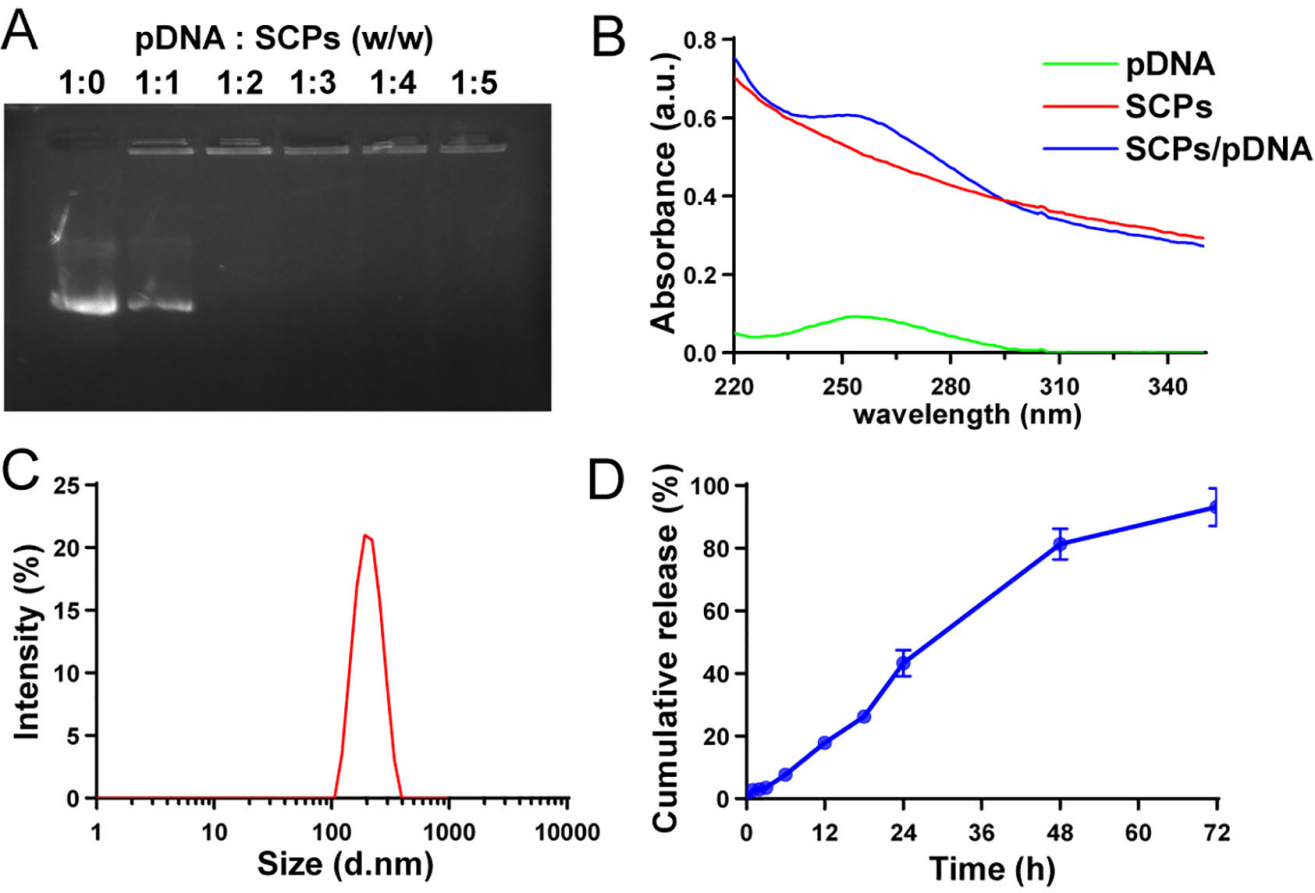
Construction and characterization of SCPs/pDNA. **(A)** Gel retardation at different weight ratios. **(B)** Size distribution analysis by DLS. **(C)** UV spectra of SCPs and SCPs/pDNA in the range of 220−350 nm. **(D)** *In vitro* release of pDNA.

The nucleic acid sequences and plasmid mapping of pHAF and pHAG are shown in **Figures 1A, B**. The agarose gel blocking results showed that pDNA was completely blocked in the upwelled wells when the ratio of pDNA to SCPs was 1:2 (**Figure 2A**). The particle size of SCPs/pDNA was measured by DLS to be ~226 nm, and the peak shapes showed a symmetrical narrow and homogeneous (**Figure 2B**). UV absorption results showed that the UV absorption level of SCPs/pDNA was significantly increased at 260 nm compared to SCPs (**Figure 2C**), indicating that pDNA was encapsulated into SCPs/pDNA. *In vitro* release data showed that the cumulative release of pDNA from SCPs/pDNA was 93.14% ± 4.12% at 72 h (**Figure 2D**), pDNA could be continuously released from SCPs/pDNA.

### 3.2 SCPs/pDNA can be transfected into 293T cells and DC2.4 cells

**Figure 3:**
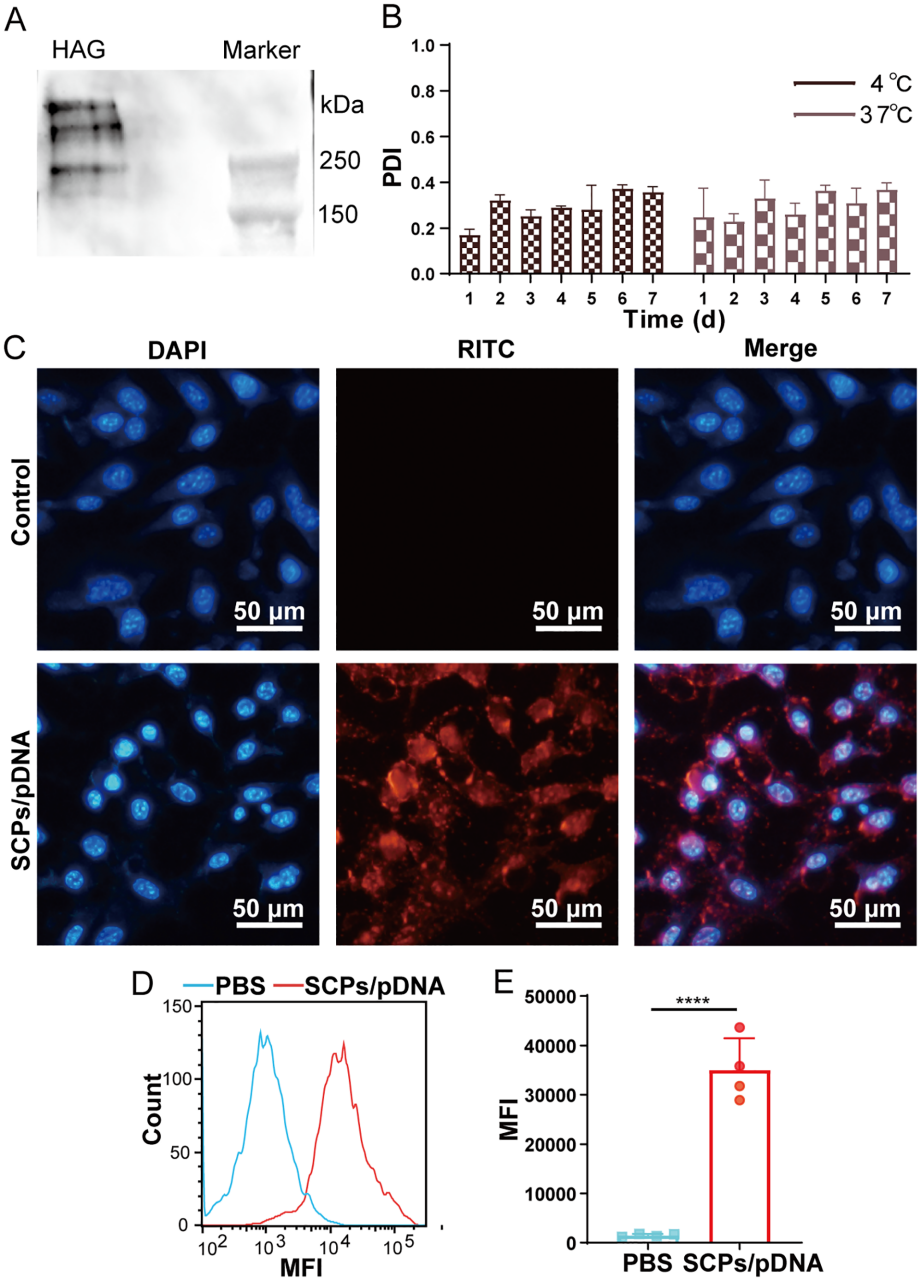
Evaluation of SCPs/pDNA. **(A)** 8% Native-PAGE Western blot analysis of expressed protein. **(B)** Stability analysis at 4°C and 37°C performed by monitoring changes in the PDI over time. **(C)** Fluorescence images of DC2.4 cells after incubation with SCPs/pDNA. **(D)** Cellular uptake profiles in DC2.4 cells by flow cytometry. **(E)** Quantification of MFI in cellular uptake assay. The data were shown as means ± SDs and statistical significance was analyzed by Tukey ‘s multiple test. ***, 0.0001<*p*<0.001.

SCPs/pDNA transfection was evaluated using SCPs/pDNA as a representative. 293T cells were transfected with SCPs/pDNA and the supernatant was collected, and the HAG protein expression was analyzed by Western blot after Native-PAGE. The results showed the presence of high molecular weight protein bands above 250 kD, which may be multimers formed by HAG self-assembly (**Figure 3A**; **Supplementary Figure S2**), confirming that pDNA in the particles can be delivered into the cells and express HA recombinant protein antigen. Stability monitoring results showed that the PDI of SCPs/pDNA could be maintained above and below 0.3 for 7 days at 4°C or 37°C, respectively (**Figure 3B**), indicating that SCPs/pDNA could remain stable under simulated storage conditions and simulated *in vivo* physiological conditions, and was suitable for refrigerated storage and *in vivo* drug delivery. Fluorescence images showed that there was an obvious RITC signal in DC2.4 cells (**Figure 3C**); the MFI value of the SCPs/pDNA group was significantly increased (*p* < 0.001) compared to the control group (**Figures 3D, E**), indicating that DC2.4 cells were able to take up SCPs/pDNA.

### 3.3 SCPs/pDNA induces humoral immunity and helper T cell immune response

**Figure 4:**
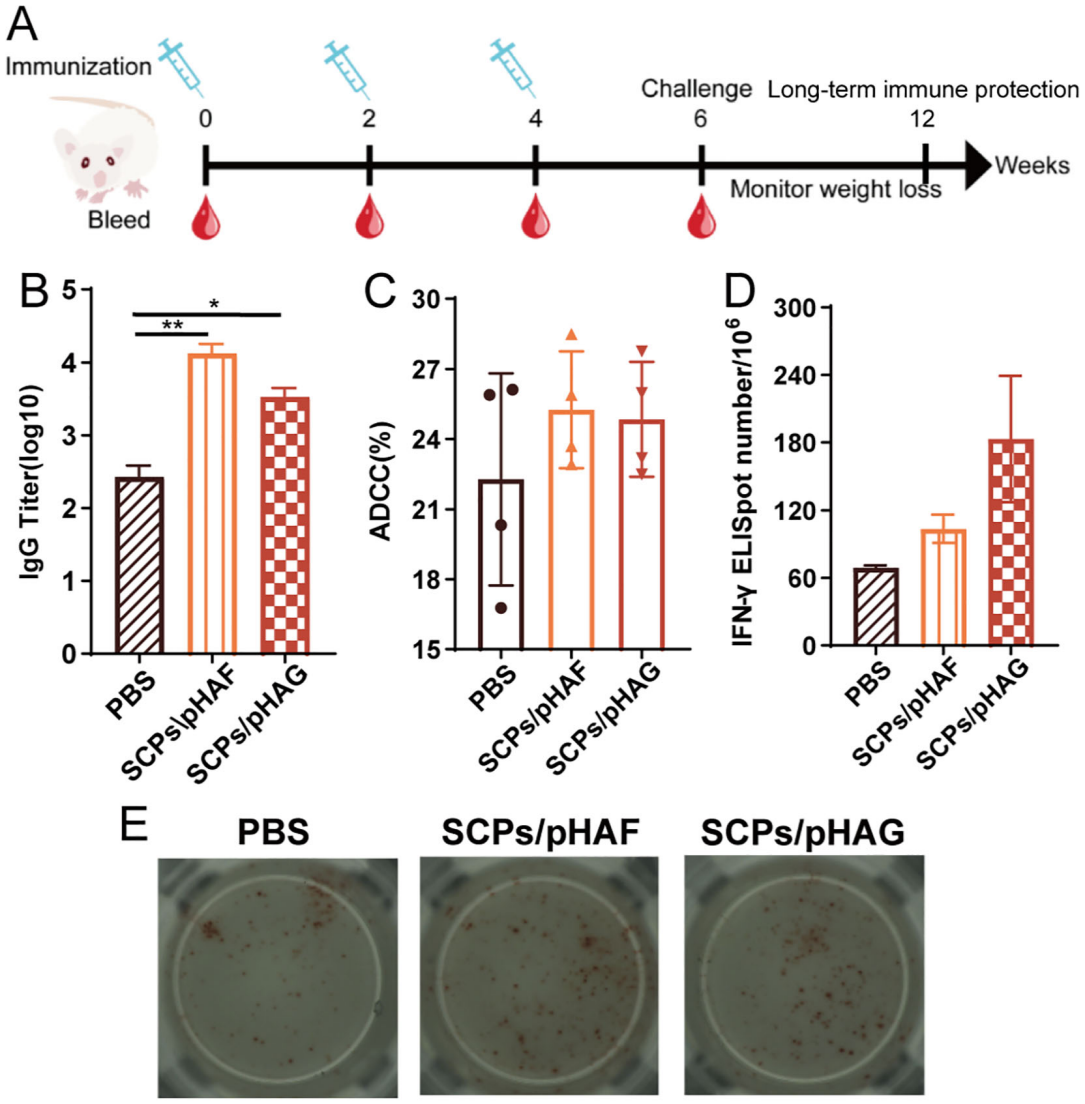
Humoral immune responses induced by SCPs/pHAF and SCPs/pHAG in mice. **(A)** Immunization and bleeding schedule of mice. **(B)** HA specific IgG titers in serum samples collected at week 6. The data were shown as means ± SDs and statistical significance was analyzed by Tukey ‘s multiple test. *, 0.01<*p*<0.05; **, 0.001<*p*<0.01. **(C)** ADCC responses in serum samples. **(D)** and **(E)** IFN-γ-secreting lymphocytes isolated from spleens were determined by ELISpot.

Mice were inoculated with SCPs/pHAF or SCPs/pHAG using a prime-boost strategy (**Figure 4A**). Upon completion of immunization, HA-specific binding antibody titers were significantly higher in the sera of mice in the SCPs/pHAF & SCPs/pHAG immunized groups compared to the control group (*, 0.01<*p*<0.05; **, 0.001<*p*<0.01) (**Figure 4B**). The results of the HA-specific ADCC assay showed that no significant nanoparticle-immunized group was observed in the ADCC response (*p* > 0.05), indicating that sufficiently significant antibody Fc-mediated cell-killing viability was not produced (**Figure 4C**). The number of cytokine-secreting cells was detected at the single-cell level by enzyme-linked immunospot assay (ELISpot), and it was found that after HA protein stimulation, there was a tendency for an increase in the number of IFN-γ spots in the SCPs/pHAF and SCPs/pHAG group compared to the control group, but there was no significant difference (*p* > 0.05) (**Figures 4D, E**). Blood was taken 2 weeks after the third immunization to determine the serum HI and MN activities, and found that there was also no significant difference (*p* > 0.05) between the SCPs/pDNA immunized groups and the control group (**Table 1**). The above results showed that SCPs/pDNA triggered significant antibody secretion with a trend toward increased ADCC and Th1 responses.

**Table 1:** HI titer and ID_50_ values of mixed serum samples against influenza A virus.

Serum Samples	A/17/California/2009/38	
	HI assay	MH assay
PBS	4	<20
SCPs/pHAF	4	<20
SCPs/pHAG	4	<20

### 3.4 SCPs/pDNA induces long-lasting and significant T cell immune responses

**Figure 5:**
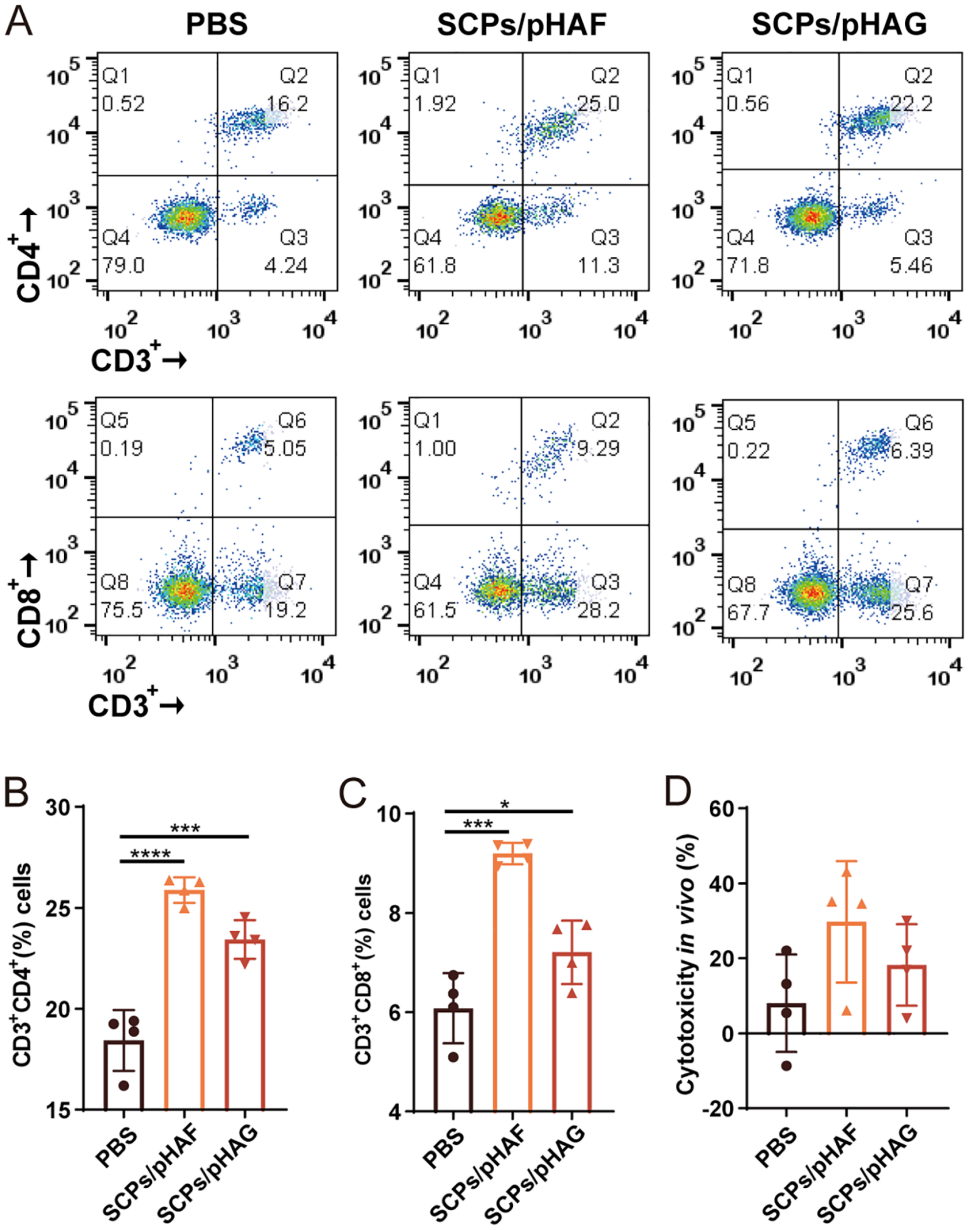
Cellular immune responses induced by DNA vaccine in mice. **(A)** Splenic lymphocytes isolated from mice immunized with nanovaccines were stained with anti-CD3^+^, CD4^+^, and CD8^+^ mABs, and analyzed by flow cytometry. **(B)** Percentage of CD3^+^CD4^+^ T cells within splenic lymphocyte population, n = 4. The data were shown as means ± SDs and statistical significance was analyzed by Tukey ‘s multiple test. ***, 0.0001<*p*<0.001; ****, *p*<0.0001. **(C)** Percentage of CD3^+^CD8^+^ T cells within splenic lymphocyte population, n = 4. The data were shown as means ± SDs and statistical significance was analyzed by Tukey ‘s multiple test. *, 0.01<*p*<0.05; ***, 0.0001<*p*<0.001. **(D)** Specific CTL responses of immunized mice, n = 4.

Flow cytometry and CTL assays demonstrated that SCPs/pHAF and SCPs/pHAG immunization induced sustained CD^4+^ and CD^8+^ T-lymphocyte proliferation (*p* < 0.05) (**Figures 5A–C**, **Supplementary Figure S1**, **Supplementary Table S1**) and enhanced CTL-mediated killing activity compared to controls (*p* > 0.05) (**Figure 5D**), indicating robust T-cell immunoreactivity in mouse spleens at 12 weeks post-immunization. The T-cell immunoreactivity was maintained for at least 12 weeks, indicating that the T-cell immunoreactivity had a long-lasting effect.

### 3.5 SCPs/pDNA protects mice from lethal infection by heterozygous strains of virus

**Figure 6:**
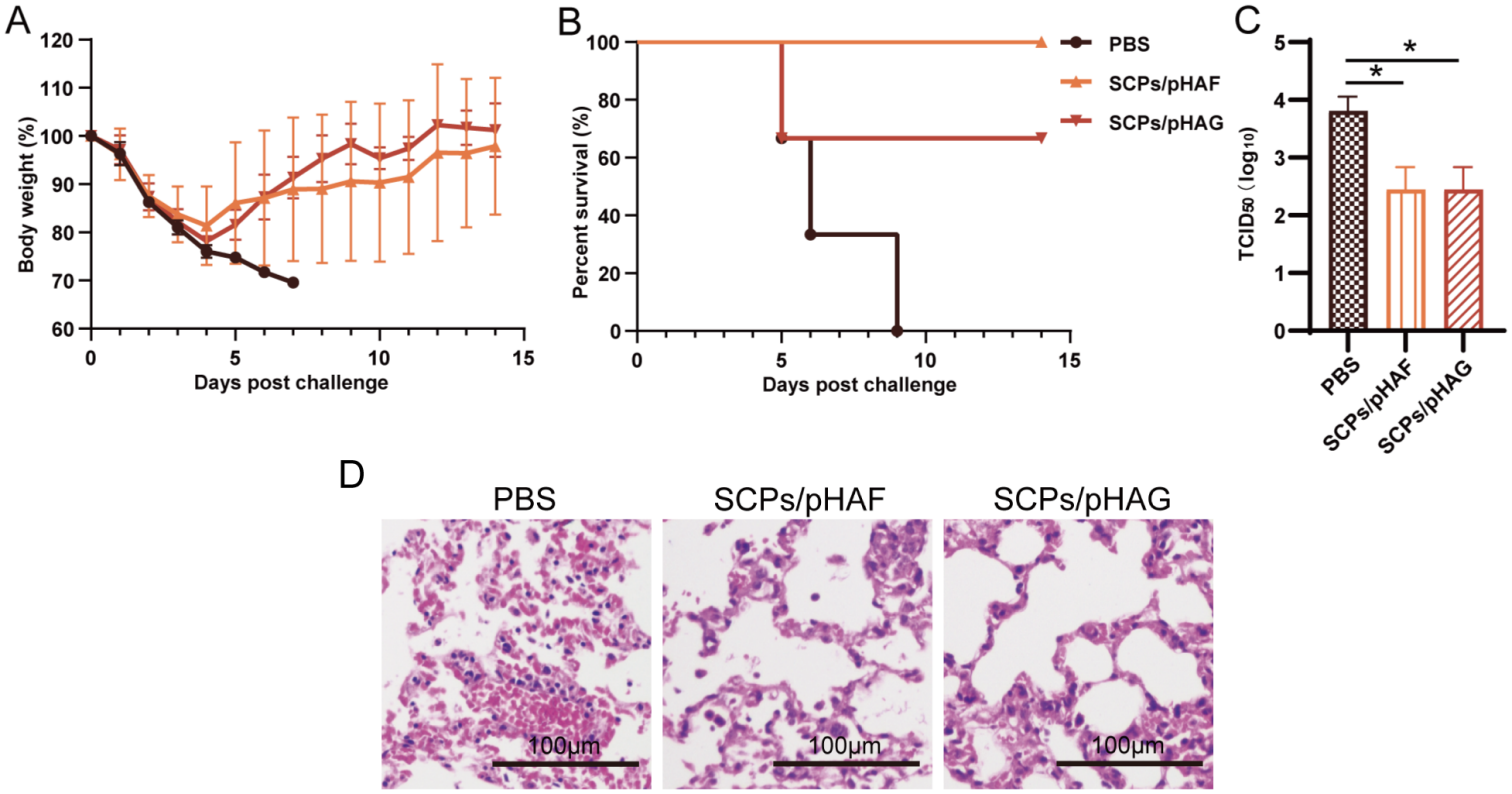
Protective efficacy against A/Hong Kong/2671/2019(H3N2) challenge. **(A)** Body weight changes and **(B)** survival curves of influenza-infected mice, n = 4. **(C)** Lung viral titers were measured 4 days after the challenge. The data were shown as means ± SDs and statistical significance was analyzed by Tukey ‘s multiple test. *, 0.01<*p*<0.05. **(D)** Lung tissues obtained from mice infected under various conditions were subjected to H&E staining.

Immunized mice were attacked using the heterozygous H3N2 strain, all mice in the control group died within 8 days of attack. One mouse died in the SCPs/pHAG group with a survival rate of 66%, and all mice in the SCPs/pHAF group survived. The body weights of mice in the immunized group all returned to the pre-attack level by day 14 (**Figures 6A, B**). The results of lung viral titer load assay showed that the lung tissue viral load of mice in the immunized group was significantly lower than that of the control group (0.01<*p*<0.05) (**Figure 6C**). The H&E results showed that the lung tissue morphology of the immunized group was more regular compared to that of the control group (**Figure 6D**). SCPs/pHAF and SCPs/pHAG induced cross-protection of mice protects mice from lethal attacks from cross-subtype viruses.

## 4 Discussion

Influenza viruses, which are prone to antigenic drift and antigenic switching, remain a serious threat to global health security. There is an urgent need for effective vaccine strategies against highly mutated influenza viruses. Nanoparticle vaccine ferritin carriers have demonstrated superior advantages in developing effective vaccines (29–32). Various nanoparticle formulations have demonstrated significant immunoenhancing capabilities, effectively boosting immune responses (33). These include polymeric nanoparticles, virus-like particles, carbon-based nanomaterials, gold nanoparticles, and lipid-based nanoparticlesn (34–36).

Vaccines designed to elicit robust T-cell-mediated immunity hold significant potential for providing cross-protection against heterologous influenza subtypes, thereby reducing viral transmission and offering a promising direction for future vaccine development. Previous studies have found that multi-epitope DNA vaccines constructed by recombining influenza virus epitopes with vectors such as Ferritin, GCN4pII, and Nov P particles all elicited strong immune responses (19, 37). In addition to SCPs-CpG, which also elicited high levels of immune responses (28) and demonstrated superior antigen delivery efficiency compared to mRNA vaccines (38). These studies reveal the potential of recombinant influenza DNA vaccines with SCPs vectors to enhance T-cell immune responses. In future studies, we will prioritize vaccine candidates that effectively enhance both humoral and cellular immunity, with an emphasis on optimizing neutralizing antibody responses.

In this study, we developed a novel multi-epitope nano-antigenic DNA vaccine using a ferritin carrier. This carrier was engineered with HA gene fragments, along with ferritin and GCN4pII sequences, which were subsequently delivered using SCPs as a novel vector to elicit potent T cell-mediated immunity. Previous studies have demonstrated that dendritic cells (DCs) can initiate a cascade of innate immune responses through IFN-γ secretion, which is widely recognized as a key cytokine driving Th1-type immune polarization (39, 40). SCPs/pDNA induced significant HA-specific binding antibodies as well as a trend toward an elevated ADCC versus Th1 response. Although the Th1-polarized immune response did not reach statistical significance, both SCPs/pHAF and SCPs/pHAG formulations effectively elicited robust antigen-specific T-cell responses. It may be because that SCPs/pDNA activate the specific immune system by triggering a Th2-type or even a Th17-type immune response. An unbalanced helper T-cell activation has also been reported in a previous study (41, 42).

Although neutralizing antibody titers were modest, this observation consistent with previous studies on DNA vaccine immunogenicity and reflects the tendency of this ferritin carrier to induce robust T-cell responses. SCPs/pDNA induced more significant CD4^+^ and CD8^+^ T lymphocyte proliferation and CTL-mediated killing activity in immunized mice even after up to 12 weeks of immunization, suggesting that SCPs/pDNA triggers a significant and long-lasting T cell immune response. Previous studies have demonstrated that pHAF and pHAG provide a high level of protection against homologous strains. In order to specifically validate the cross-protective capacity of T-cell immunity, the present study chose to directly attack the virus with heterozygous strains. The immune response triggered by the H1N1 antigen protected mice from lethal H3N2 infection, and the fact that the two strains were not on the same phylogenetic tree (5), indicated that SCPs/pDNA induced a certain level of cross-protection and increased the resistance of mice to the cross-subtype virus.

It has been shown that many factors may influence the size and morphology of calcium phosphate precipitates (43), and that calcium phosphate particles are usually composed of a mixture of phases of different calcium phosphates (44). The observed heterogeneity in SCPs/pDNA complexes may influence their uptake efficiency by antigen-presenting cells (APCs) and subsequent humoral immune activation. Nevertheless, the inherent stability of SCPs/pDNA enables sustained antigen delivery, facilitating the induction of robust and durable T-cell-mediated immune responses. Furthermore, CD^4+^ T cells play a pivotal role in both the initiation and maintenance of immunological memory, orchestrating the development of B cell-mediated humoral immunity and CD^8+^ T cell-mediated cellular immunity (45, 46). This strongly suggests that CD^4+^ T cells may exert direct protective effects in response to influenza. The precise mechanisms underlying their protective effects remain to be elucidated: whether they mediate direct antiviral activity *in vivo*, recruit additional host immune cells through rapid cytokine and chemokine production, or confer protection via a synergistic combination of these mechanisms.

The present study demonstrated that SCPs/pDNA complexes could induce robust and durable T-cell immune responses in mice, while significantly enhancing their resistance to cross-subtype lethal viral challenges. These findings highlight the promising potential of SCPs/pDNA-based carriers as novel delivery systems for next-generation influenza vaccines. In contrast to previous studies on SCPs-CpG/pDNA (28), and the use of Toll-like receptors (22, 47), polysaccharides (48) or ATP (49) to modify the surface of CPs for the development of novel adjuvants, it is foreseen that a diverse range of immune agonists could be similarly utilized for modification of SCPs/pDNA to obtain even more promising vaccine-delivery vector alternatives.

## 5 Conclusion

In summary, SCPs/pDNA triggered a significant T-cell immune response. This T-cell immune response can be maintained for at least 12 weeks and has certain longevity. The cellular immunity significantly reduces pulmonary viral loads in mice, indicating broad cross-protective efficacy against lethal challenge with heterologous viral strains. From studies about T-cell immunity triggered by SCPs/pDNA combined with Toll-like receptor-functionalized calcium phosphate nanoparticles, we may foresee that the delivery of influenza DNA vaccines with SCPs vectors alone or in combination with Toll-like receptor may induce balanced humoral and cellular immune responses with long-lasting cross-protective effects.

## Data Availability

The original contributions presented in the study are included in the article/**Supplementary Material**. Further inquiries can be directed to the corresponding author.

## Supplementary material

Supplementary Figure 2 12% SDS-PAGE of expressed HAG proteins.
