# Supplementary material for: Silica-calcium phosphate nanoparticles delivering recombinant influenza hemagglutinin DNA can induce long-lasting T cell immune cross-protection in mice
Source: Front Immunol. 2025 Jun 3;16:1572618. doi: 10.3389/fimmu.2025.1572618 (PMC12188357; doi:10.3389/fimmu.2025.1572618)
Supplement: Supplementary Figure 2 — 12% SDS-PAGE of expressed HAG proteins. [file DataSheet2.docx]

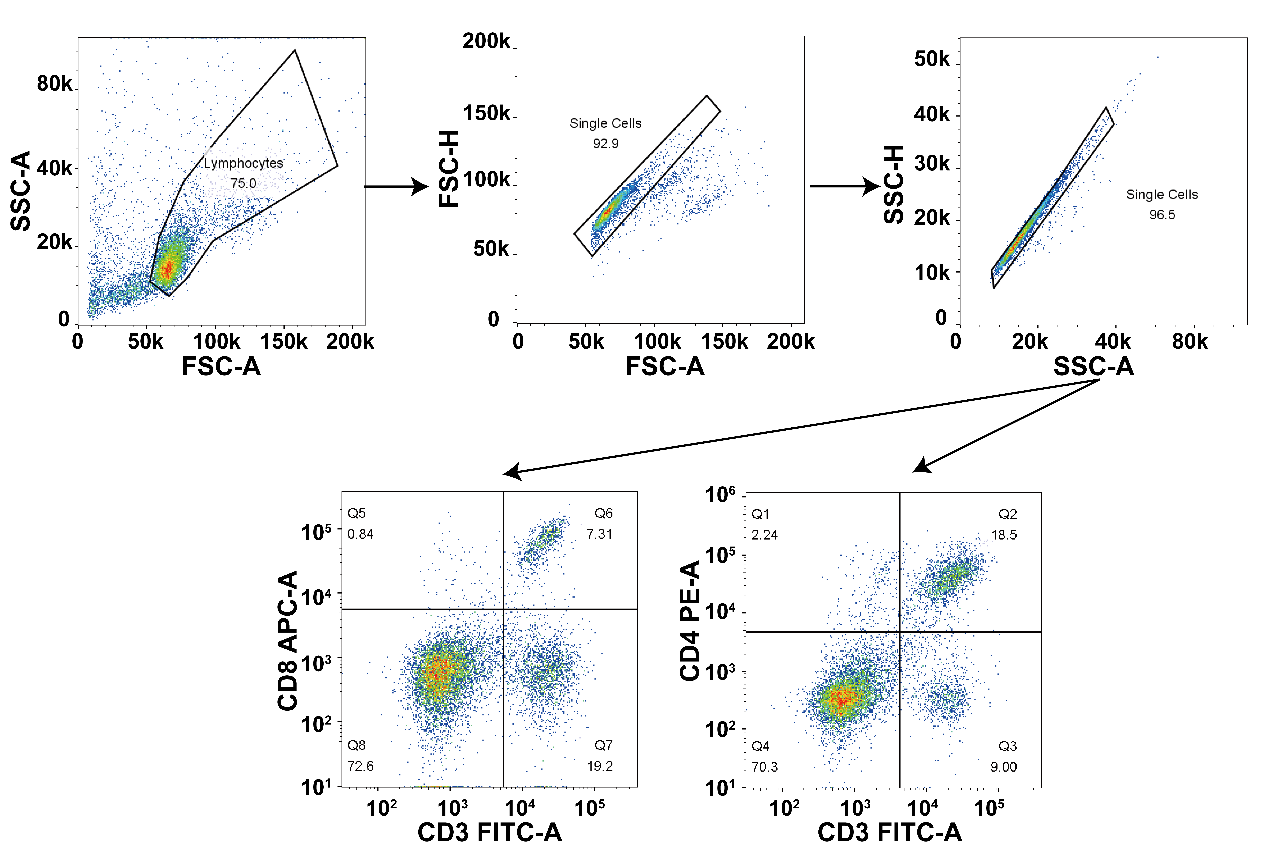


Figure S1. The gating strategy used for the flow cytometry. Lymphocytes were gated based on forward scatter area (FSC-A) and side scatter area (SSC-A). A forward scatter height (FSC-H) vs. FSC-A density plot can be used to exclude doublets. A side scatter height (SSC-H) vs SSC-A plot can also be used. Furthermore, live CD3 positive T cells were then further identified and gated by the expression of CD4 and CD8 to identify helper and cytotoxic cells.


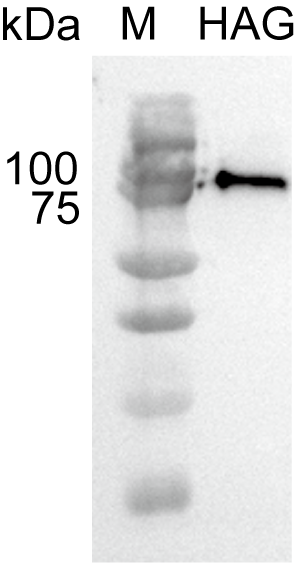


**Figure S2.** 12% SDS-PAGE of expressed HAG proteins.

Table S1. Antibodies used for flow cytometry.

| Target | Vendor | Concentration |
| --- | --- | --- |
| CD4 | BioLegend | 0.5 µg per million cells in 100 µL volume |
| CD3 | BioLegend | 0.5 µg per million cells in 100 µLvolume |
| CD8 | BioLegend | 0.5 µg per million cells in 100 µL volume |
| CD16/32 | BioLegend | 0.25 µg per million cells in 100 µL volume |
